# Rethinking Antigen Source: Cancer Vaccines Based on Whole Tumor Cell/tissue Lysate or Whole Tumor Cell

**DOI:** 10.1002/advs.202300121

**Published:** 2023-05-31

**Authors:** Lu Diao, Mi Liu

**Affiliations:** ^1^ Department of Pharmaceutics College of Pharmaceutical Sciences, Soochow University 199 of Ren ai Road Suzhou Jiangsu 215123 P. R. China; ^2^ Kunshan Hospital of Traditional Chinese Medicine Kunshan Jiangsu 215300 P. R. China; ^3^ Suzhou Ersheng Biopharmaceutical Co., Ltd. Suzhou 215123 P. R. China

**Keywords:** cancer vaccines, immunotherapy, neoantigens, tumor specific antigens, whole tumor lysates

## Abstract

Cancer immunotherapies have improved human health, and one among the important technologies for cancer immunotherapy is cancer vaccine. Antigens are the most important components in cancer vaccines. Generally, antigens in cancer vaccines can be divided into two categories: pre‐defined antigens and unidentified antigens. Although, cancer vaccines loaded with predefined antigens are commonly used, cancer vaccine loaded with mixed unidentified antigens, especially whole cancer cells or cancer cell lysates, is a very promising approach, and such vaccine can obviate some limitations in cancer vaccines. Their advantages include, but are not limited to, the inclusion of pan‐spectra (all or most kinds of) antigens, inducing pan‐clones specific T cells, and overcoming the heterogeneity of cancer cells. In this review, the recent advances in cancer vaccines based on whole‐tumor antigens, either based on whole cancer cells or whole cancer cell lysates, are summarized. In terms of whole cancer cell lysates, the focus is on applying whole water‐soluble cell lysates as antigens. Recently, utilizing the whole cancer cell lysates as antigens in cancer vaccines has become feasible. Considering that pre‐determined antigen‐based cancer vaccines (mainly peptide‐based or mRNA‐based) have various limitations, developing cancer vaccines based on whole‐tumor antigens is a promising alternative.

## Graphical abstract

Three components are critical in cancer vaccines: antigen, adjuvant, and formulation. Among them, antigen is the most essential component in cancer vaccine. Selecting antigens is the most important step in designing vaccines and cancer cell is the best antigen source for preparing cancer vaccines. Herein, cancer vaccines based on cancer cells or cancer cell lysates are reviewed.

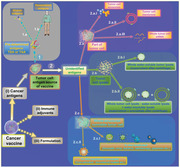

## 1 Introduction

Cancer immunotherapy has been tried to be applied in clinical use for a long time, since 1893.^[^ ^1^ ^]^ Normally, cancer immunotherapy functions through enhancing or normalizing the immune system of the cancer patient to fight against the cancers and boosting or rescuing the antitumor immune responses. Different types of cancer immunotherapy strategies have been extensively studied and certain success in terms of clinical effects have been achieved.^[^ ^2^, ^3^ ^]^ Cancer immunotherapy has brought great hope to the fight against cancer in recent years, owing to the improved survival in a certain proportion of patients and the induction of long‐term immune memory. During the last two decades, several immunotherapy approaches, such as check point inhibitors and T cell‐mediated adoptive therapies, have been used against solid and hematological malignancies. Among these immunotherapy methods, cancer vaccine is a critical approach to prevent and treat various cancers.^[^ ^4^ ^]^

T cells, especially tumor antigen‐specific T cells, are the main killers of cancer cells, and most cancer immunotherapy rely on the activation of T cells, either through the re‐stimulation of the inhibited pre‐existing T cell clones or enlarging the T cell repertoire.^[^ ^5^ ^]^ Modifying T cells in vitro to express certain tumor‐specific antigens enables achieving precise targeted therapy, such as chimeric antigen receptor T‐cell immunotherapy (CAR‐T). CAR‐T therapy certainly offers new promise for cancer treatment. However, CAR‐T has not shown strong efficacy in the treatment of solid tumors, and sometimes it has been associated with some serious side effects, mainly cytokine release syndrome (also known as cytokine release storm, CRS) and neurotoxicity.^[^ ^6^, ^7^, ^8^ ^]^ It can be life‐threatening if left untreated. Therefore, CAR‐T cells must be closely monitored after infusion. The check point inhibitors, such as PD‐1 monoclonal antibodies and CTLA‐4 monoclonal antibodies, work through reversing the suppression of pre‐existing T cell responses.^[^ ^9^ ^]^ Several PD‐1 monoclonal antibodies and CTLA‐4 monoclonal antibodies have been approved by FDAs in different countries. The checkpoint inhibitors show remarkable therapeutic efficacy and have changed the treatment of cancer. However, some patients remain refractory to these approaches and their objective response rates rarely exceed 15–30%.^[^ ^10^, ^11^, ^12^ ^]^ Therefore, it is essential to develop novel methods to overcome these limitations, and cancer vaccine is a potential candidate that would change the treatment of cancer in future.

**Figure 1:**
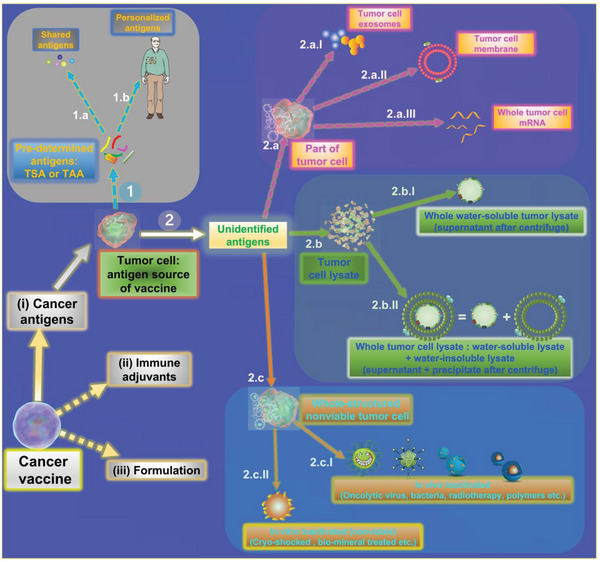
Schematic illustration of the source of tumor antigens to prepare cancer vaccines.

The efficacy of cancer vaccines mainly depends on cellular immunity and the vaccines efficiency of activation of antigen‐specific T cells.^[^ ^13^ ^]^ Two steps are crucial for activating tumor antigen‐specific T cells: step 1 is the efficient uptake by antigen‐presenting cells (APCs), especially dendritic cells (DCs) and the efficient antigen presentation by APCs; step 2 is the efficient activation of the naïve T cells by the antigen‐presented APCs.^[^ ^14^ ^]^ In order to develop an effective cancer vaccine, it is crucial that several parameters be optimized and such parameters include: antigens, adjuvants, and formulation. The tumor antigens are the most important and the determining parameter in developing a potent cancer vaccine, and it is essential to utilize optimized tumor antigens for developing cancer vaccines.^[^ ^15^, ^16^, ^17^ ^]^ Therefore, antigen selection is the most important step in the design of tumor vaccine. Different types of cancer antigens that could be used to prepare cancer vaccines are summarized in **Figure** **1**. Ideal antigens for cancer vaccines should have several properties, such as: 1) high immunogenicity; 2) inclusion of pan‐spectra (all kinds or at least most kinds) of tumor‐specific antigens; 3) specificity; 4) easy and fast to obtain and so on. Tumor cell/tissue is the best antigen source for developing cancer vaccines. Generally, we can classify tumor antigens into two types: pre‐defined neoantigens and undefined mixed antigens (anonymous). Both these tumor antigens could be derived from tumor cells/tissues.

Cancer cells are recognized by the immune system through unique tumor specific antigens (TSAs), also called neoantigens.^[^ ^18^ ^]^ Therefore, it is crucial to include such antigen sources in cancer vaccines. However, due to the high heterogeneity and complexity of cancer cells, most neoantigens are personalized.^[^ ^19^, ^20^ ^]^ To date, very limited shared antigens have been identified in cancers, and most of these shared antigens are insufficient for preparing universal vaccines due to the heterogeneity and complexity of the tumor antigens.^[^ ^21^ ^]^ Different methods for preparing cancer vaccines based on neoantigens (mainly peptide‐based neoantigens, mRNA‐based neo‐antigens or DNA‐based neoantigens), either individualized neoantigens or shared neoantigens, have been explored.^[^ ^22^ ^]^ The development of neoantigen‐based vaccines requires the determination and identification of the neoantigen before the development of the cancer vaccine.^[^ ^23^ ^]^ Therefore, the preparation is complicated and time‐consuming. For instance, in case of the individualized peptide‐based neoantigen: the collected sample tumor should be sequenced and the neoantigen identification performed using computational tools (considering factors such as predictive proteasome processing and MHC class‐I and‐II binding affinities) and mass spectrometry, followed by custom synthesis of the identified neoantigens; the customized neoantigens are mixed with adjuvants and administered to patients.^[^ ^24^ ^]^

Neoantigen‐based cancer vaccines have been widely tested in clinical trials. Since around 10 years ago, several clinical trials of neoantigen cancer vaccines (both peptide‐based vaccines and mRNA‐based vaccines) have been investigated in different clinical trial phases.^[^ ^25^ ^]^ However, no neoantigen‐based vaccine has been approved owing to the requirement of pre‐determined antigens.^[^ ^26^ ^]^ This limitation includes but is not limited to: 1) the high heterogeneity of neoantigens and the rarity of neoantigens; 2) it is time‐consuming (several months) to prepare neoantigen‐based vaccines; 3) the high heterogeneity and diversity of cancer cells and tumor antigens; 4) the inaccuracies in neoantigen identification;^[^ ^27^, ^28^, ^29^ ^]^ 5) immunogenicity of antigens is not positively correlated with the abundance of neoantigens; 6) the high heterogeneity of different cancer cells; 7) the efficacy is limited due to the difficulties in covering the neoantigens of most cancer cells by using 10−30 neoantigens; 8) the cost could be very high and the process is very complicated.^[^ ^20^, ^27^, ^28^, ^29^, ^30^, ^31^ ^]^

Considering that the clinical efficacy of tumor vaccines is hindered by the lack of broadly‐expressed tumor antigens in several cancers and the not sufficiently accurate pre‐determinations of neoantigens, using the tumor cells or tumor tissues (either autologous or allogeneic), without pre‐determining and identifying neoantigens, for developing cancer vaccines seems promising.^[^ ^32^, ^33^ ^]^ Using the tumor cells or tumor tissues, without pre‐determining and identifying neoantigens, can enable the development of polyclonal individualized cancer vaccines.^[^ ^34^ ^]^ These would address the lack of universally expressed tumor antigens and the risk of immune escape with treatments that target single or a few antigens.^[^ ^35^ ^]^

Several approaches could be used for preparing tumor‐cell‐based cancer vaccines: whole tumor cells (the tumor cell are nonviable but not destroy the structure of tumor cells), tumor cell lysate (the tumor cell are nonviable and destroy the structure of tumor cells) and some components or compartments of tumor cells.^[^ ^36^ ^]^ In case of tumor cell lysate, only the water‐soluble components from the whole cell lysate were used in previous studies for preparing cancer vaccines. Recently, Liu's group showed that both water‐soluble and solubilized water‐insoluble components can be encapsulated into cancer vaccines, owing to the technology they developed.^[^ ^20^, ^37^ ^]^ Whole tumor antigen vaccines offer a relatively simple approach to circumvent some of these limitations by including a broad array of TSAs that contain epitopes for both CD8^+^ cytotoxic T cells (CTLs) and CD4^+^ T helper cells. Autologous whole tumor cell‐based vaccines with presentation of both MHC Class I and II restricted antigens would help generate a stronger overall anti‐tumor response and long term CD8^+^ T cell and CD4^+^ T cell immune memories.^[^ ^38^ ^]^ In indications where surgery can be performed as part of treatment, autologous cancer cells isolated from the resected tumor tissue can be used as a source of neoantigens to be loaded in cancer vaccines, considering that “self” antigens in the cancer cells have already induced immune tolerance.^[^ ^39^, ^40^ ^]^ Live tumor cells are poorly immunogenic; they secrete or contain factors that could inhibit DC and T cell function. Therefore, methods to inactivate the cancer cells and prepare whole cancer neoantigens are critical to produce cancer vaccines with high immunogenicity.^[^ ^41^ ^]^

Tumor antigens in cancer vaccines cannot activate T cells directly. The antigens should be taken up by antigen‐presenting cells (APCs) and then presented through them for T cell activation. APCs, especially dendritic cells (DCs), are critical mediators in the activation of T cells. Therefore, the formulation of cancer vaccines could be based on tumor antigens directly or based on tumor antigen stimulated DCs. Antigen immunogenicity is critical for the efficient induction of T cell responses. Several approaches have been explored to improve the immunogenicity of tumor antigens, which in turn will improve the efficacy of cancer vaccines.

Tumor‐specific antigens, selected from tumor cells, have been studied and developed through various methods. Several reviews have summarized the studies on pre‐determined‐neoantigen‐based cancer vaccines.^[^ ^42^ ^]^ Therefore, in this review, we focus on the advances of cancer vaccines based on unidentified (anonymous) antigens, especially those based on the cancer cell/tissue lysates. We have first summarized the recent developments in cancer vaccines that use a part of the tumor cells, such as membrane, exosomes, or whole mRNA. Then, we describe the past success and current efforts in developing cancer vaccines based on whole tumor cells without lysing cells. At the end, we have discussed the recent progress in developing cancer vaccines based on tumor cell/tissue lysate, especially based on whole tumor cell/tissue lysate including both water‐soluble components and solubilized water‐insoluble components. During the discussion, the methods to improve the immunogenicity of tumor antigens are also discussed at the relevant places. Both vaccines formulation, based on tumor antigens directly and based on tumor antigen stimulated DCs, are discussed

## 2 Cancer Vaccines Loaded with Part of Whole Tumor Antigens

Exosomes, whole RNA, and membrane of cancer cells contain part of the antigens in whole tumor antigens. They do not have all the immunogenic antigens in cancer cells; however, such cancer vaccines show preventive and therapeutic efficacy in various cancers.^[^ ^34^, ^43^ ^]^

### 2.1 Cancer Vaccines Based on Tumor Exosomes

**Figure 2:**
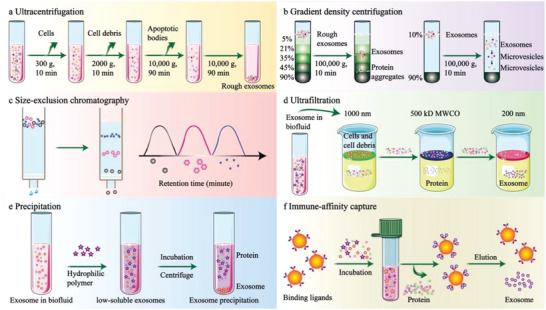
Conventional exosome isolation methods. a) Ultracentrifugation, b) gradient density centrifugation, c) size‐exclusion chromatography, d) ultrafiltration, e) precipitation‐based method, f) immunoaffinity‐based method.

**Figure 3:**
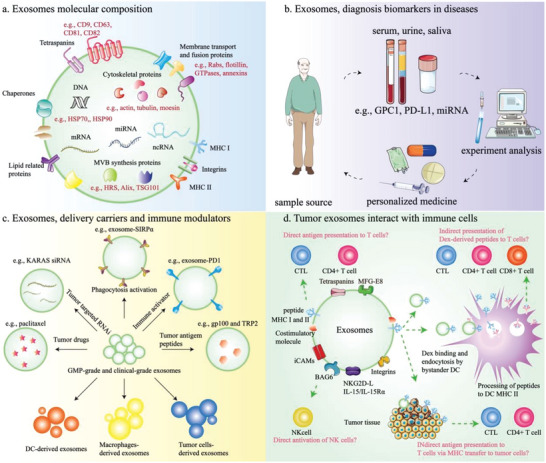
Exosomes in cancer immunotherapy. a) Exosomes contain a complex mixture including proteins, mRNA, miRNA, ncRNA, and DNA. Exosome surface proteins, such as CD9, CD63, and CD81 can be used as indicators for identification. The proteins TSG101 and Alix are involved in the formation of internal vesicles of MVBs. b) Exosomes with GPC1, PD‐L1, or certain miRNA derived from cancer patients could be valuable as cancer biomarkers. c) Exosomes have high stability in circulation and good capacity to transfer horizontal cargo; they have been explored as delivery carriers loaded with small molecule drugs; they can target tumor RNA and proteins in various cancers. The exosomes secreted from immune cells such as DCs and macrophages and tumor cells stimulate specific anti‐tumor immune responses. d) The presence of MHC I and MHC II molecules on the surface of tumor exosomes gives them the potential to directly stimulate CTLs and CD4+ T cells. Tumor exosomes can also activate T cells indirectly by presenting antigens through DC cells.

Exosomes are membrane‐enclosed extracellular vehicles (EVs) ranging from 40 to 160 nm in diameter; they originate from the endosomal compartment, also referred to as “small EVs” in a subset of the literature.^[^ ^44^, ^45^ ^]^ Methods for the isolation of exosomes have been studied extensively (**Figure** **2**). Exosomes contain functional biomolecules (proteins, lipids, RNA, and DNA) that can be transferred horizontally to recipient cells (**Figure** **3**a). Tumor‐derived exosomes (TEXs) are emerging as regulators of tumorigenesis.^[^ ^46^, ^47^ ^]^ The exosomes carry molecular information about the parent tumor. There is increasing evidence that exosomes are agents of cell‐to‐cell local and systemic communication; therefore, they can transfer functional substances to recipient cells or participate in membrane receptor‐mediated signaling in recipient cells.^[^ ^48^ ^]^ Mounting evidence suggests that exosomes could be used for early cancer detection, prognosis, and to guide therapy. A study reported a surface plasmon response (SPR)‐based assay for label‐free, high‐throughput exosome protein analyses, which improved sensitivity over previous methods, enabling portable operations when integrated with miniaturized optics and allowing the retrieval of exosomes for further study.^[^ ^49^, ^50^ ^]^

Nano‐plasmonic exosome assay is used for analyzing ascites samples from ovarian patients. The exosomes from ovarian cancer cells can be identified by their expression of CD24 and EpCAM, suggesting the potential of exosomes for cancer diagnostics or therapy.^[^ ^51^, ^52^ ^]^ The discovery of proteins that are abundant in exosomes and could be isolated from the cells of origin is a critical milestone in the field. To pave way for an unbiased exploration of ubiquitous and abundant exosome biomarkers in a high‐resolution and quantitative manner, Raghu et al. developed a quantitative proteomic approach based on labeling amino acids with a super‐stable isotope, for high‐resolution mass spectrometry.^[^ ^53^ ^]^ These studies provided more information about the exosome tumor antigens that could be used in cancer vaccines (Figure 3b).

Over the past 10 years, the studies on exosomes increased rapidly due to discoveries that exosomes play key roles in the occurrence and development of tumors.^[^ ^54^ ^]^ Exosomes from a highly metastatic melanoma increased the metastatic behavior of primary tumors by permanently “educating” bone marrow progenitors via the MET receptor. Melanoma‐derived exosomes also induce vascular leakage at the pre‐metastatic site and reprogram BM progenitor cells into a C‐Kit+Tie2+Met+ pre‐angiogenic phenotype.^[^ ^55^ ^]^ Exosomes of tumor origin are very important for tumor development and metastasis. Therefore, some researchers have wondered whether knocking out certain proteins on exosomes that are derived from tumors could inhibit metastasis signaling. Poggio et al. discovered that removal of exosomal PD‐L1 inhibits tumor growth; this was an alternative mechanism of PD‐L1 activity involving its secretion in tumor‐derived exosomes. The exosomes derived from PD‐L1‐deficient tumor cells suppressed the growth of wild‐type tumor cells. Anti‐PD‐L1 antibodies are useful in cases with tolerance issues. Tumor‐derived exosomes could be a new therapeutic target, which could overcome the challenges of current antibody approaches.^[^ ^56^, ^57^ ^]^

DC‐derived exosomes form a new class of vaccines for cancer immunotherapy. Exosomes derived from tumor antigenic peptide‐pulsed DCs (DEXs) elicit strong immune responses and tumor suppression in mammary carcinoma mice.^[^ ^58^, ^59^ ^]^ DEXs have been tested in clinical trials on advanced melanoma patients, and they show encouraging results. In addition, DEXs show promising results in hepatocellular carcinoma (HCC). Exosomes derived from *α*‐fetoprotein‐expressing DCs are isolated using high‐speed ultracentrifugation and 0.22 µm diafiltration to stimulate antigen‐specific immune responses in HCC models. They induce strong antigen‐specific immune responses and tumor retardation in ectopic and orthotopic HCC mice^[^ ^60^, ^61^ ^]^ (Figure 3c).

Vesicles that are secreted by living tumor cells contain and transfer tumor antigens to DCs. After exogenous uptake of mouse tumors, dendritic cells induce effective CD8+ T cell‐dependent antitumor effects on allogeneic mouse tumors. Therefore, exosomes represent a new type of tumor rejection antigen derived from T‐cell cross primers that are associated with immune intervention.^[^ ^62^ ^]^ TEXs are emerging as regulators of tumorigenesis. Melanoma‐secreted exosomes reprogram bone marrow progenitor cells in the premetastatic niche, to convert them to an angiogenic phenotype. Specific proteins present in the exosomes determine organ‐specific metastasis and the survival of patients with cancer.^[^ ^63^ ^]^ In addition, TEXs convey activation or inhibitory molecular signals to immune cells, during which they directly or indirectly influence cell development, maturation, and antitumor activity. TEXs can transfer activated epidermal growth factor receptor (EGFR) to host macrophages, suppressing innate antiviral immunity. TEXs antagonize innate antiviral immunity in an EGFR‐ and MEKK2‐dependent manner.^[^ ^64^ ^]^ DCs pulsed by HCC cell lysates were moderately effective; the response rate was marginal and the efficacy was low. Exosomes from tumor cells can efficiently deliver a variety of tumor antigens to DCs, and they have been employed as antigen carriers for cancer immunotherapy. HCC exosomes‐pulsed DCs induced a significantly stronger immune response and suppressed tumor growth in HCC mice compared to DCs pulsed with cell lysates.^[^ ^65^, ^66^ ^]^ The tumor immune microenvironment was significantly improved in HCC mice treated with TEX‐pulsed DCs; they showed increased number of T lymphocytes, elevated levels of IFN‐*γ*, and decreased levels of IL‐10 at the tumor site.^[^ ^67^ ^]^

Tumor‐secreted microRNAs bind to murine TLR7 and human TLR8 in immune cells to trigger tumor metastasis. The role of tumor exosomal RNA in tumorigenesis is studied extensively. Lung epithelial cells are critical for initiating neutrophil recruitment, and the lung metastatic niche is formed by sensing tumor exosomal RNAs via Toll‐like receptor 3 (TLR3).^[^ ^68^, ^69^ ^]^ Identification of the metastatic axis of tumor exosomal RNAs and host lung epithelial cell TLR3 activation provides potential targets against cancer metastasis to the lung.^[^ ^70^ ^]^ Not only tumor‐derived exosomes, but normal cell‐derived exosomes can also be engineered as vectors to target cancer genes. For example, exosomes that are derived from normal fibroblast‐like mesenchymal cells were engineered to carry short interfering RNA or short hairpin RNA specific to oncogenic Kras, a common mutation in pancreatic cancer. Treatment using engineered exosomes (known as iExosomes) show suppression in multiple mouse models of pancreatic cancer and a significant increase in overall survival.^[^ ^61^, ^71^ ^]^ However, the lack of a standardized process for the isolation of exosomes with high purity and the documented risk of inducing immunosuppressive effects have hampered clinical applications (Figure 3d).

### 2.2 Cancer Vaccines Based on Whole Tumor RNA

Cancer vaccines loaded with whole tumor RNA can be developed as DC vaccines or nanocarrier form vaccines. We have summarized the recent advances in both areas.

#### 2.2.1 DCs Pulsed/Enveloped with Whole Tumor RNA

Using tumor total RNA (ttRNA) for the introduction of TSAs is one of the strategies for the improving DC vaccines. In essence, ttRNA offers all the possibilities offered by total tumor lysates for “personalizing” a therapy to individual patients, but with a few additional benefits.^[^ ^72^ ^]^ In contrast to total tumor lysates, ttRNA requires minimal sample volume of the tumor and is not renewable upon depletion. DCs transfected with ttRNA can express known tumor antigens, and these antigen‐expressing DCs can activate CTLs against the original tumors. This strategy has been used in a phase I clinical trial in renal cell carcinoma, newly diagnosed and recurrent pediatric brain tumors, prostate cancer, and melanoma.^[^ ^73^ ^]^ The use of tumor RNA‐pulsed DCs was reliable in expanding CD4+ and CD8+ tumor‐reactive T lymphocytes, for curative adoptive cellular therapy in a highly‐invasive, chemotherapy‐ and radiation‐resistant malignant glioma model.^[^ ^74^ ^]^ Transfection of DCs with whole cell RNA is more effective than that with DNA vector constructs. The first RNA‐transfected‐DC‐based clinical study indicated that this form of vaccination is feasible and safe. Ovalbumin (OVA) is one of the most common tumor model antigens, which can be delivered by nanoparticles to antigen‐presenting cells in tumors or lymph nodes. The pioneering experiments demonstrated that DCs pulsed with total mRNA of OVA can induce OVA‐specific immune response to kill tumor cells expressing OVA proteins (such as B16F10‐OVA). Tumor RNA and tumor lysates have been used as a source of whole tumor antigens to prepare dendritic cell (DC)‐based tumor vaccines. Comparisons have been made to identify which antigen formation is the most effective. Coukos et al. showed that DCs electroporated with tumor cell RNA induced a higher tumor infiltration by T cells and produced a significantly stronger delay in tumor growth compared to that with DCs pulsed with UV‐irradiated tumor cells.^[^ ^75^ ^]^ In addition, pulsing DCs with whole tumor RNA showed better results compared to that with fusing DCs with autologous tumor cells.^[^ ^76^ ^]^

Several reports have indicated that loading DCs with whole tumor mRNA is an effective nonviral strategy to stimulate T cell responses both for in vitro and in vivo models.^[^ ^77^, ^78^ ^]^ In 2005, researcher demonstrated the method of using DCs that are transfected with RNA coding for whole tumor RNA to induce potent antigen specific T cell responses in detail.^[^ ^79^ ^]^ Scientists described the basic techniques for the successful genetic modification of DCs by using the mRNA electroporation method.^[^ ^51^ ^]^ DCs electroporated with whole tumor RNA stimulate antigen‐specific T cells in vitro and induce antigen‐specific T‐cell responses in melanoma patients vaccinated with DCs electroporated with full‐length TAA‐encoding mRNA. All possible antigenic epitopes of the TAA will be presented instead of some selected epitopes.^[^ ^80^ ^]^ This strategy was applied to acute myeloid leukemia; it resulted in the induction of leukemia‐specific cytotoxic T lymphocytes and the identification of B1‐1 peptide as a novel immunogenic tumor‐associated antigen.^[^ ^81^ ^]^ Mice with CT26 tumors were immunized with DCs co‐transfected with GM‐CSF mRNA and CT26 whole tumor mRNA; the cytotoxic activity of immunized mice was significantly higher than that of the control group.^[^ ^64^ ^]^ Mu et al. reported that among 19 patients with androgen‐resistant prostate cancer that were treated with total tumor‐derived mRNA loaded DC vaccines, 8 patients progressed, 11 patients were considered to have stable disease, and 13 patients showed a decrease in serum prostate‐specific antigen levels.

#### 2.2.2 Nanotechnology Used to Deliver Whole Tumor RNA

The use of whole tumor RNA is considered promising and potentially effective. To improve the inherent RNA stability, nanocarriers, which are nano‐drug delivery systems, are developed. Total tumor‐derived RNA extracted from liver cancer cells (Hepa1‐6 cells) were encapsulated in lipid nanoparticles (LNPs) and delivered to target DCs; this stimulates expeditious and robust anti‐tumor immunity.^[^ ^82^ ^]^ Kranz et al. encapsulated RNA encoding tumor antigens into liposomes; these target DCs and activate tumor‐specific T cell immune effects. Several RNA‐liposome cancer vaccines are undergoing human clinical trials, and they have shown encouraging early results. To improve whole tumor mRNA delivery and in vivo protein expressions, liposomes, and polymeric micelle‐based formulations are under development, including nanoparticle delivery vehicles made from lipid‐like materials.^[^ ^83^, ^84^ ^]^ Anderson et al. developed compounds of ionizable lipid‐like materials for the use in mRNA delivery vehicles that facilitate mRNA delivery in vivo and provide potent and specific immune activation, inhibit tumor growth, and prolong survival in melanoma.^[^ ^82^, ^85^, ^86^ ^]^ This strategy is also effective for spontaneous malignant gliomas. Whole tumor RNA (derived from whole tumor cell transcriptome) are encapsulated in lipid‐NPs with excess positive charge; this primes the peripheral and intratumor milieu for response to immunotherapy. These personalized tumor whole mRNA‐NPs were safe and active in a client‐owned canine with a malignant glioma.^[^ ^87^ ^]^ The positive results in phase I clinical trials have demonstrated that whole tumor RNA vaccines are safe and immunogenic in patients with melanoma and gastric cancer, with encouraging clinical outcomes. In addition, vaccines based on whole tumor DNA are studied extensively.^[^ ^88^ ^]^ Chen et al. set up a DNA‐based neoantigen vaccine platform containing antigens identified through comprehensive identification of individual somatic mutations using whole tumor‐exome sequencing, DC‐based vaccine, optimization of DNA vaccine nanocarrier, and combination with adjuvants (CpG); it achieved a significant tumor regression in a melanoma mouse model and considerably inhibited lung metastases in another mouse model.^[^ ^89^ ^]^

### 2.3 Cancer Vaccines Based on Tumor Cell Membrane

Antigen epitopes will be processed and presented on the tumor cell membrane. In addition, some immunogenic proteins are located on the surface of the membrane. Therefore, tumor cell membranes could be used as the antigen source for preparing cancer vaccines.^[^ ^90^ ^]^ Some such studies are reviewed here.

#### 2.3.1 Tumor Cell Membrane Enveloped Bacterial/Oncolytic Virus Membrane

Tumor cell membranes contain a high proportion of antigenic motifs and have been proposed for use in the development of tumor vaccines. However, the immunogenicity of membrane antigens is weak and the immunosuppressive molecules present on the cell surface block recognition by the immune system.^[^ ^91^ ^]^ Nie et al. developed an antigen and adjuvant codelivery nanoparticle vaccine using *Escherichia coli* cytoplasmic membranes and tumor cell membranes from resected autologous tumor tissue to generate polylactic acid‐hydroxy acetic acid (PLGA) nanoparticle vaccines (HM‐NPs). The personalized tumor vaccine was designed to enhance the safety of innate immune responses, maximize the antitumor effects, while avoiding side effects.^[^ ^92^ ^]^ Bacteria that contain various microbe‐associated molecular patterns are the most common immunostimulatory agents. The vesicles (mTOMV) constructed using bacterial outer membrane vesicle are hybridized with the cell membrane that originated from the tumor tissue. This accumulates in the inguinal lymph nodes, strengthens the activation of innate immune cells, and increases the specific lysis ability of T cells in homogeneous tumors.^[^ ^93^ ^]^ Immunotherapy using tumor cell membrane enveloping oncolytic virus is very useful and popular. Viruses are recognized as non‐autologous; therefore, they initiate immune responses owing to their natural adjuvant properties. Oncolytic viruses could be artificially wrapped with cancer membranes that carry tumor antigens. In different murine tumor models, inhibition of tumor growth and activation of specific anti‐tumor responses were observed fat both therapeutic and vaccination set‐up stages. This was achieved with the help of a membrane extrusion process during which the cell membrane was successfully coated with viruses.^[^ ^94^ ^]^ Ideally, after the trachomatous injection of oncolytic viruses, sufficient number of tumor antigens are released and collected by DCs to present to T cells in the lymphoid organs, ultimately leading to an antitumor immune response.

#### 2.3.2 Tumor Cell Membrane‐Coated Lipid/PLGA Nanoparticles

Among the different bioinspired strategies, using the cellular membrane material in nanoparticles represents a unique top‐down approach that offers the advantage of completely replicating the surface antigenic diversity of source cells.^[^ ^95^ ^]^ For example, RBC membrane coated nanoparticles have long‐circulation properties, stem cell‐derived membrane coated ones have cancer targeting capabilities, and leukocyte membrane coated ones have the ability to traverse endothelium.^[^ ^96^ ^]^ Monophosphoryl lipid A (MPLA), a toll‐like receptor 4 (TLR4) agonist, is employed in human trials for the development of a malarial vaccine.^[^ ^97^ ^]^ Liposomes are food and drug administration (FDA)‐approved nanomaterial; they present a phospholipid bilayer structure, which is similar to the structure of the cell membrane. Some studies have constructed a biohybrid liposome by reconstituting MPLA, 4T1 cell membranes, and some common lipids.^[^ ^98^ ^]^ The vaccine triggered and enhanced bone marrow dendritic cell maturation and central memory T cells (TCM) differentiation.^[^ ^99^ ^]^ Cell‐derived nanoparticles have been garnering increased attention due to their ability to mimic many of the natural properties displayed by their source cells. Researchers reported the biological functionalization of polymeric nanoparticles with a layer of membrane coating derived from cancer cells together with immunological adjuvants. These core‐shell nanostructures carry the full array of cancer cell membrane antigens and offer a robust platform with applications in multiple modes of anticancer therapy.^[^ ^100^ ^]^

The ideal cancer vaccine should contain strong immunogenic cancer‐specific antigens and effective adjuvants to stimulate strong cellular immunity; this is key to clearing cancer cells. The coating of tumor cell membranes on nanoparticles combined with CpG not only carried tumor antigens for specific anti‐tumor immunity but also increased the dispersion and stability of NPs, promoting their targeting to lymph nodes for the efficient activation of T cells.^[^ ^101^, ^102^ ^]^ PEGylated nanoparticles extend circulation time in the body. Qiu et al. derived endogenous cell membranes from cancer cells and added them to PEGylated nano‐vesicles, which exhibited good serum stability and draining efficiency to local lymph nodes following subcutaneous administration.^[^ ^103^ ^]^ In tumor‐bearing mice, inoculation of PEG‐NPs synthesized by mouse melanoma cells resulted in a 3.7‐fold higher antigen specific cytotoxic CD8+ T lymphocyte response than that with standard vaccine that contained freeze‐thawed lysate.^[^ ^104^ ^]^ The bionic membrane nano‐vaccine (CCMP@R837), which is composed of 4T1 cancer cell membrane, coated on PLGA nanoparticles and loaded with imiquimod (R837) as an adjuvant to activate the immune system, has achieved good efficacy in the treatment of breast cancer.^[^ ^105^ ^]^ Gu et al. reported a cancer vaccine using erythrocyte membrane and aPDL1 blockers that utilize damaged RBCs to deliver tumor‐associated antigens (TAAs) to key secondary lymphoid organs; this effectively enhanced melanoma and breast cancer immunotherapy on mouse model.^[^ ^106^ ^]^

Tumor cell membrane modified with mannose can enhance DCs uptake and cause a stronger stimulation effect for triggering DCs maturation.^[^ ^107^ ^]^ The surface of tumor cell membranes can be modified to improve the tumor targeting. For instance, a tumor cell line can be engineered to express the co‐stimulatory marker CD80 to target CD28 on T cell surface. The membrane from these engineered cells can be coated onto a nanoparticulate substrate, resulting in a biomimetic nanoformulation that can direct antigen presentation to cancer‐specific T cells, this nanoformulation inhibit tumor growth in triple negative breast mouse cancer models.^[^ ^108^ ^]^ However, these approaches are only promising when the tumor tissue is available in sufficient quantity. When the amount of tumor sample is limited, it is essential to use fewer tumor membranes to activate a stronger immunity. A DC‐based vaccine, in which DCs are pulsed with tumor membrane vesicles (TMVs) and incorporated with glycolipid‐anchored immunostimulatory molecules (GPI‐ISMs) were tested in HER2‐positive and triple negative breast cancer murine models. TMVs that contain GPI‐GM‐CSF and GPI‐IL‐12 improved DCs activation and inhibited tumor growth.^[^ ^109^ ^]^ DCs are the most effective antigen presenting cells for inducing T cell response. The only FDA approved DC‐based immunotherapy to date is Sipuleucel‐T, which was stimulated in vitro with GM‐CSF using a fusion protein and delivered with the prostate cancer antigen, PAP. Small interfering RNA (siRNA) can be modified to inhibit the expression of certain genes when given as vaccines, it has a remarkable effect on the treatment of melanoma in mice.^[^ ^110^ ^]^ A DC‐targeted nanovaccine has been proposed; it includes the phospholipid bilayer from tumor cell membrane and imiquimote (IMQ), a clinically approved TLR‐7 agonist. IL‐10 siRNA loaded into NVs inhibit IL‐10 secretion during TLR agonist‐mediated DCs maturation. The nanovaccine surface was further modified with an FC‐binding peptide and then non‐covalently bound with anti‐CD205 antibody to enhance the ability of DCs to target the CD205 receptor. The vaccine is highly protective in melanoma models.^[^ ^111^ ^]^

#### 2.3.3 Tumor Cell Membrane‐Based Superparamagnetic/Photothermal Vaccine

Cell membrane coated nanoparticles that integrate the biophysiological advantages of cell membranes with the multifunctionalities of synthetic materials hold great promise in cancer immunotherapy.^[^ ^112^ ^]^ To enable more precise delivery of tumor vaccine to the lymph nodes to activate DCs, a novel cancer vaccine was developed by using Fe_3_O_4_ magnetic nanoclusters as the core and cancer cell membranes embedded with anti‐CD205 as the covering layer.^[^ ^113^ ^]^ This design enabled us to magnetically retain them in the lymph nodes under the guidance of magnetic resonance imaging (MRI). The adjuvant CpG, which is regarded as a TLR agonist can also be adsorbed with the vaccine. The results showed that a large amount of T cells proliferated with great clonal diversity and superior cytotoxic activity.^[^ ^113^ ^]^ Pd‐l1 inhibitory peptide (TPP1) and MMP2 substrate peptide (PLGLLG) have been conjugated to the tumor cell membrane (SPIO NP@M‐P) to construct superparamagnetic iron oxide nanoparticles, which effectively extend the half‐life of the peptides and maintain their ability to reactivate T cells and inhibit tumor growth.^[^ ^114^ ^]^ Zhang et al. developed the nanoengager, which consists of an NIR absorbing polymer as the photothermal core, camouflaged with fused membranes derived from immunologically engineered tumor cells and DCs as the cancer vaccine shell. Photothermal therapy has been integrated with cancer immunotherapy because PTT can induce ICD to enhance anti‐cancer immunity.^[^ ^115^ ^]^ Using a combination of phototherapy and chemotherapy shows synergistic effects in tumor therapy. The co‐loading of curcumin and chlorin e6 (Ce6) into PLGA nanoparticles and coating them with MCF‐7 cell membranes was a promising strategy in mice breast cancer.^[^ ^116^ ^]^ Thermosensitive hydrogel‐coated cytokines have been used as the core in the design of personalized tumor‐specific vaccines. A personalized vaccine that was ingeniously designed used a surgically removed tumor for preparing a photothermal vaccine and combined it with antibody programmed death‐1 (PD‐1) to prevent tumor relapse and metastasis. Black phosphorus quantum dot nanovesicles (BPQD‐CCNVs) coated with cell membranes of surgically resected tumors were prepared and loaded into a thermosensitive hydrogel that containing GM‐CSF and lipopolysaccharide (LPS).^[^ ^117^ ^]^ Sustained release of GM‐CSF and LPS from the hydrogel could recruit and activate DCs. Taken together, personalized cancer vaccines show great potential in cancer immunotherapy by inducing an effective and durable antitumor response.^[^ ^118^ ^]^

#### 2.3.4 Tumor Cell Membrane‐Based Novel Probe/Material Vaccine

**Figure 4:**
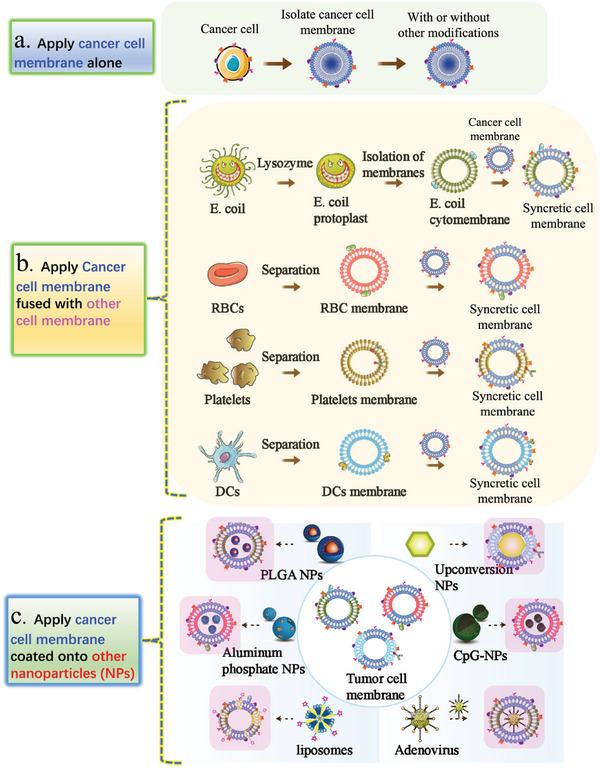
The strategy of cancer cell membrane‐based vaccines. a) Applying tumor cell membrane alone. b) Applying hybrid cell membranes fused with cancer cell membrane. c) Applying nanoparticles (NPs) coated with cancer cell membrane.

Cancer metastases and recurrence following surgical resection remains as an important cause of treatment failure. Discovering new materials to promote high levels of antigen cross‐presentation and to elicit high levels of antitumor T‐cell responses is also a research hotspot.^[^ ^119^ ^]^ Different types of surface engineering approaches have been developed for modifying the nanoparticles with either artificial materials or natural substances.^[^ ^120^, ^121^ ^]^ Liu et al. observed that a fluoropolymer could be employed for fabricating personalized cancer vaccines by combining it with tumor cell membranes collected from surgically resected autologous tumors. This fluoropolymer‐based personalized nanovaccine can prevent postoperative tumor recurrence and metastasis when combined with ICB. This simple and reliable strategy to manufacture cancer nanovaccines that are based on fluoropolymers promises to be an effective patient‐specific postoperative immunotherapy.^[^ ^122^ ^]^ Genomic instability is one of the most common characteristics of tumor cells and it could be targeted for the treatment of tumors. A composite system was derived from branched glycopolymer‐pyropheophorbide, and it was further modified with tumor cell membranes and was detectable via magnetic resonance imaging (MRI). The mechanism underlying this biomimetic nanomedicine is that it could be irradiated to produce ROS that induces DNA damage and prevents DNA repair to kill tumor cells. The design concept of this novel material will provide a new idea for the development of bioinspired synergistic nanodrugs based on weak interactions^[^ ^123^ ^]^ (**Figure** **4**). Molecular imaging of nanomedicine is critical in the diagnosis and treatment of tumors. Molecular imaging can, in principle, provide powerful tools for identifying cancers with greatly improved specificity and sensitivity. In addition to the well‐known roles of enhancing tumor penetration and retention of nanoobjects, functional inorganic nanoparticles show great potential as imaging probes, due to their inherent physical properties.^[^ ^124^, ^125^ ^]^ Hou et al. developed a novel probe based on cancer cell membrane‐coated upconversion nanoparticles, which successfully differentiated MDA‐MB‐231 tumor models through in vivo modality imaging, which could be used for breast cancer molecular classification^[^ ^126^ ^]^ (Figure 4).

## 3 Cancer Vaccines Based on Whole Tumor Cell (Without Destroying the Cell Structures)

Whole tumor cell contains all tumor antigens; therefore, it is good antigen source to prepare cancer vaccines. Researchers have developed methods to apply whole tumor cells as antigens in cancer vaccines. Such kind of cancer vaccines retain the structures of tumor cells.^[^ ^127^, ^128^ ^]^ Therefore, antigens need to be released from the tumor cells to be uptaken and processed by APCs.

### 3.1 Cancer Vaccine Based on Cryo‐Shocked Whole Tumor Cell

Cryotherapy, also known as hypothermia, is one of the physical therapies for cancer. To treat tumors, a machine that produces ultra‐low temperatures rapidly is used to cool the diseased part such that the diseased tissue degenerates, undergoes necrosis, and finally falls off. It is a new medical technology that has developed rapidly in the treatment of benign and malignant tumors.^[^ ^129^, ^130^ ^]^ Freezing can preserve tissues under certain conditions, but can them under the others. The biological cell death caused by freezing is a comprehensive result of several factors. Liquid nitrogen is the most widely used refrigerant in cryotherapy at present. It has a low boiling point (−196 °C), is safe to use, and is widely available.^[^ ^131^ ^]^ The freezing time depends on the method of freezing and the size of the tumor, ranging from 30 s to 30 min. A duration of 15 min can achieve 80–90% of the maximum freezing effect. At present, cryotherapy is mainly used in clinical setup for superficial tumors that are easy to contact directly, such as skin, head and neck, facial features, rectum, cervix, and prostate. In recent years, cryotherapy for visceral tumors such as liver, lung, and kidney are also being actively explored.^[^ ^132^ ^]^

Dead tumor cells left by liquid nitrogen‐based cryo‐shocking can be used as a drug‐targeting carrier and tumor vaccine for cancer therapy. Gu et al. used a liquid nitrogen‐based cryo‐shocking method to obtain therapeutic dead cells.^[^ ^133^ ^]^ These cells maintained the intact structure, allowing for drug encapsulation, but had lost their proliferation ability and pathogenicity. Specifically, cryo‐shocked acute myeloid leukemia (AML) cells retained the homing capability of their bone marrow and served as a drug delivery vehicle of doxorubicin (DOX), which is a critical drug used in the induction chemotherapy against AML.^[^ ^134^ ^]^ When used in combination with chemotherapy, cryo‐shocked AML cells stimulated an immune response to eliminate leukemia in tumor mice. Preimmunization with LNT cells and adjuvants protected healthy mice from AML cell attack. This kind of “dead cell”‐based delivery vehicle can be rapidly manufactured for clinical use compared to live cell‐based drug delivery systems.^[^ ^133^ ^]^ Recently, the effect of combining cryo‐treatment and immunotherapy was reported.^[^ ^135^ ^]^ In these basic and clinical studies, CTLA‐4 and PD‐1 antagonists play complementary roles in activating cancer immunity, and cryotherapy after immunotherapy can induce a stronger cancer‐specific immune response to distant lesions. Based on these findings, the abstract effect of re‐implantation of liquid nitrogen‐treated tumor‐bearing bone grafts and the synergistic effect of anti‐PD‐1 therapy were evaluated using a bone metastasis model. The number of CD8+ T cells infiltrating the abscopal tumor and tumor‐specific IFN‐*γ*‐producing spleen cells increased in the liquid nitrogen‐treated group.^[^ ^136^ ^]^ Liquid nitrogen‐based cryo‐shocking of tumor cells can also be used in combination with adjuvants, as vaccines against lung cancer. Tadao found that the injection of MS‐Ap‐PAMA adjuvant in combination with liquid‐nitrogen‐treated tumor tissue (derived from Lewis lung carcinoma cells) into C57BL/6 mice markedly inhibited in vivo tumor recurrence and the development of rechallenged tumor, compared to those with commercial alum adjuvant.^[^ ^137^ ^]^ More than half of the patients with metastasis liver disease die from metastatic complications. In cryoablation, liquid nitrogen or argon gas is delivered to the liver tumor, guided by ultrasound using a specially designed probe. Ice crystal formation during the rapid freezing process causes destruction of the cellular structure and kills the tumor cells.^[^ ^138^ ^]^

**Figure 5:**
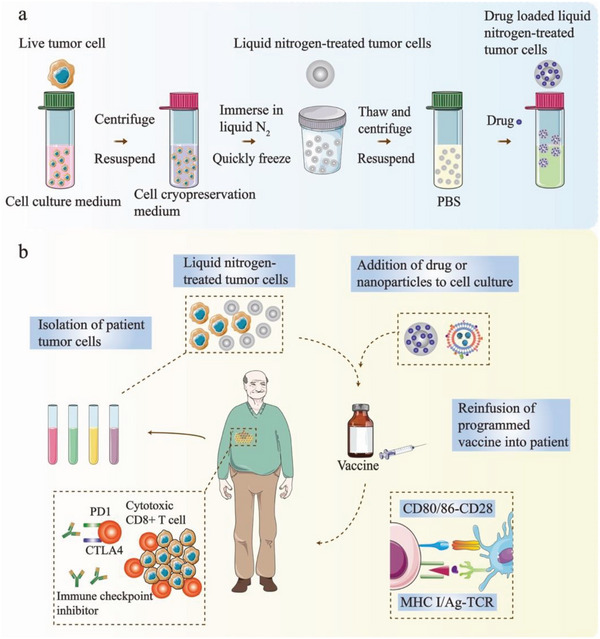
Cryo‐shocked personalized whole tumor vaccines dramatically improve effector responses. a) The procedure to prepare liquid nitrogen‐treated tumor cells and load them with drugs or nanoparticles. b) Patient tumor cells were collected, frozen using liquid nitrogen, and incubated with drug or nanoparticles. The engineered vaccine is then reinfused into the patient to activate DCs for antigen presentation. The vaccine can be combined with immune checkpoint inhibitors to amplify these antitumor T cell responses.

Colorectal tumor tissue was surgically resected and cryopreserved in liquid nitrogen to detect protein markers on the tumor surface, which can be used for preoperative detection.^[^ ^139^ ^]^ Patel et al. analyzed gene expression profiles in 68 stage I and 15 borderline ovarian cancers to determine whether different clinical characteristics (such as tissue type, grade, and survival) of stage I ovarian cancer were associated with differences in gene expression. Tumors were obtained directly during surgery and immediately frozen in liquid nitrogen until analysis.^[^ ^140^ ^]^ Lutgendorf et al. identified gene regulatory pathways that may lead to these changes using transcriptome driven, promoter‐based bioinformatics analyses.^[^ ^141^ ^]^ Frozen liquid nitrogen is also widely used in cryosurgery surgery to kill cancer cells and has been successfully used in the treatment of malignant tumors by medical experts in Omsk, Russia. The technology has brought good news to more than 50 cancer patients. The new technology works by pouring liquid nitrogen into the cancerous area using an application device, at a temperature of −196 °C, causing massive necrosis in the frozen area. The technique is currently used as an adjunct treatment for lung cancer.^[^ ^138^, ^142^ ^]^ Clinical trials have been ongoing for three years, using the technique in more than 50 patients with lung cancer. The spread of malignant tumor in the lung was effectively controlled in the treated patients, and there were no cases of recurrence or metastasis. This technique is also effective during surgery in patients with osteosarcoma. Doctors at Henan Cancer Hospital collected bone from osteosarcoma patients, stored it in liquid nitrogen at −176 °C, froze the cancer cells, and then implanted them back in to the patients.^[^ ^143^ ^]^ In 2005, Tsuchiya et al. described reconstruction using tumor‐bearing autografts treated with liquid nitrogen. Malignant bone tumors were frozen with liquid nitrogen at −196 °C, and the tumor cells were inactivated by inducing ice crystal formation and cell dehydration. Liquid nitrogen sterilized bone has superior bone induction properties than high temperature‐sterilized bone because of better preservation of bone morphogenetic proteins.^[^ ^144^, ^145^ ^]^ There have been some cases on cryosurgery applications in the oral region. In particular, oral mucosa is an ideal site for this technique because of its humidity and smoothness^[^ ^146^ ^]^ (**Figure** **5**).

### 3.2 Cancer Vaccine Based on Gel‐Encapsulated Cancer Cells

A crucial challenge in preparing cancer vaccines is the inclusion of extensive tumor antigens and overcoming the lack of co‐stimulatory signals.^[^ ^147^ ^]^ To include the whole tumor cell and the co‐stimulatory signals, Mooney et al. designed a tumor cell‐loaded cryogen sponge that could function as an injectable vaccine platform, delivering antigen‐carrying tumor cells along with GM‐CSF and a specific TLR agonist CpG, while creating space for DCs infiltration and trafficking.^[^ ^148^ ^]^ They encapsulated GM‐CSF, a DCs enhancement factor, and CpG ODN, a DCs activating factor, into a sponge‐like macroporous cryogel. These cryogels were injected subcutaneously into mice to localize in the transplanted tumor cells and deliver immunomodulatory factors in a controlled spatiotemporal manner. These cryogels can be compressed to a fraction of their original volume, and they can return to their original shape following injection. Within this material, the transplanted tumor cells have a high viability immediately following injection, and the cryogels release immunomodulatory factors in a localized and sustained manner. This whole‐tumor cell vaccine platform provided a local immunogenic niche in which the encounter of DCs and tumor cells is tightly controlled, facilitating the induction of a potent, durable, and specific anti‐tumor T‐cell response in a melanoma model. Altogether, these findings indicate the potential of cryogels as a platform for cancer vaccines.^[^ ^149^ ^]^

### 3.3 Cancer Vaccines Based on Bio‐Mineral Treated Whole Cancer Cells

Selected neoantigens are highly immunogenic, and they are used for preparing personalized cancer vaccines. Although these approaches show promise, the clinical efficacy is confined and the clinical applications have been limited owing to the lengthy and complex production requirements.^[^ ^150^ ^]^ Considering that the clinical efficacy of cancer vaccines based on pre‐determined neoantigens is limited due to the lack of broadly expressed tumor antigens among cancer cells and the risk of immune escape in treatments targeting single antigens, autologous tumor cells are used for preparing individualized tumor vaccines for inducing polyclonal T cell responses.^[^ ^151^ ^]^

Applying whole‐cancer cells as cancer vaccines could help overcome the limitations of cancer vaccines based on several selected neoantigens. However, when applying whole‐cancer cell as cancer vaccines, it is necessary to ensure that all cancer cells are nonviable. Biomineralization technology could be applied to prepare such cancer vaccines.^[^ ^152^ ^]^ Serda from the University of New Mexico, used cell cryo‐silicification technology for preparing cancer vaccines. They overcame the hurdles to create stable biomineralized tumor cells that function as a modular vaccine for the development of a highly effective personalized immune therapy. They showed that cancer vaccines can be made via the cryogenic silicification of tumor cells, which preserves tumor antigens within nanoscopic layers of silica, followed by the embedding of the silicified surface with pathogen‐associated molecular patterns.^[^ ^153^ ^]^ These pathogen‐mimicking cells could be uptaken by dendritic cells and presented as tumor antigens to T cells. In a mouse model of ovarian cancer, a cell‐line‐based silicified cancer vaccine induced tumor‐antigen‐specific T‐cell immunity and efficiently controlled tumor growth. In addition, silicified and surface‐modified cells derived from tumor samples are amenable to dehydration and room‐temperature storage without loss of efficacy and could be conducive to developing individualized cancer vaccines across tumor types.^[^ ^154^ ^]^ These results indicated that preparing cancer vaccines using biomineral technology is promising. However, the procedure to prepare such vaccines is complicated, and the potential toxicities should be systematically investigated further.

## 4 Cancer Vaccines Based on Tumor Cell Lysate

Antigens in cancer vaccines based on whole tumor cells without destroying the structure of tumor cells, need to be released from the tumor cell structures, and then uptaken and processed by APCs.^[^ ^155^ ^]^ However, antigens in cancer vaccines based on tumor cell lysates, do not require to be released from the cancer cells and can be uptaken directly, processed, and presented in the APCs.^[^ ^29^, ^156^ ^]^

### 4.1 Cancer Vaccines Based on Water‐Soluble Components in Tumor Cell Lysate

**Figure 6:**
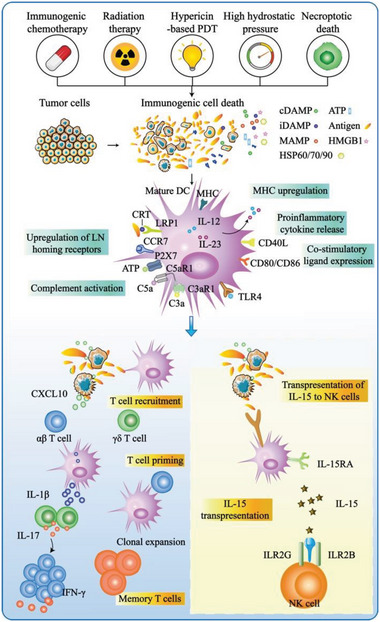
Differential requirements for the Immunogenicity of cell death and cell death pathways are upstream drivers of adaptive immunity. a) Several processes have been linked to the immunogenicity of cell death, including the unfolded protein response and consequent exposure of calreticulin (CALR) and other endoplasmic reticulum (ER) chaperones on the cell surface, the activation of autophagy, and consequent secretion of ATP and HMGB1. These factors, by binding to low‐density lipoprotein receptor‐related protein 1 (LRP1), P2×7, and Toll‐like receptor 4 (TLR4), at the dendritic cell (DCs) surface, increase antigen uptake, processing, and DCs maturation as well as upregulate major histocompatibility complex (MHC) molecules, co‐stimulatory ligands, and inflammatory cytokines. Upregulation of C–C chemokine receptor 7 (CCR7) promotes the homing of these dendritic cells to the lymph node (LN), where T cell initiation occurs. Complement activation further enhances the DCs maturation process by releasing C3a and/or C5a via alternative pathways. Mature DCs carrying tumor‐specific antigens can activate T cells to kill tumors and form specific immune memory, as well as activate NK cells by releasing IL‐15 and binding to IRL receptors on NK cells.

Water‐soluble components in tumor cell lysates are most commonly used to prepare cancer vaccines, both DC‐formed vaccines and other formed vaccines (such as free water‐soluble cell lysate added with adjuvants).^[^ ^157^ ^]^ In this part, we summarize the progress in developing cancer vaccines based on water‐soluble components in tumor cell lysates and the methods applied to improve their immunogenicity (**Figure** **6**).

#### 4.1.1 Cancer vaccines Based on Tumor Lysates Prepared through Necrosis and Apoptosis

Therapeutic vaccines are activated through one of the following: previously validated cancer antigen, peptides, or recombinant proteins from previously validated cancer antigen, or whole tumor cells or lysates containing whole antigen sources. Peptides and recombinant proteins induce a strong and potent immune response against designed immunogenic targets, but the immune escape of cancers could reduce the long‐term benefits of one or several kinds of immunogenic antigens.^[^ ^158^ ^]^ Cancer cells contain all the tumor antigens; therefore, they could potentially induce pan‐spectra tumor‐specific T cells, which makes the immune escape of cancer more difficult. Therefore, the application of whole tumor lysates is a promising approach.

**Figure 7:**
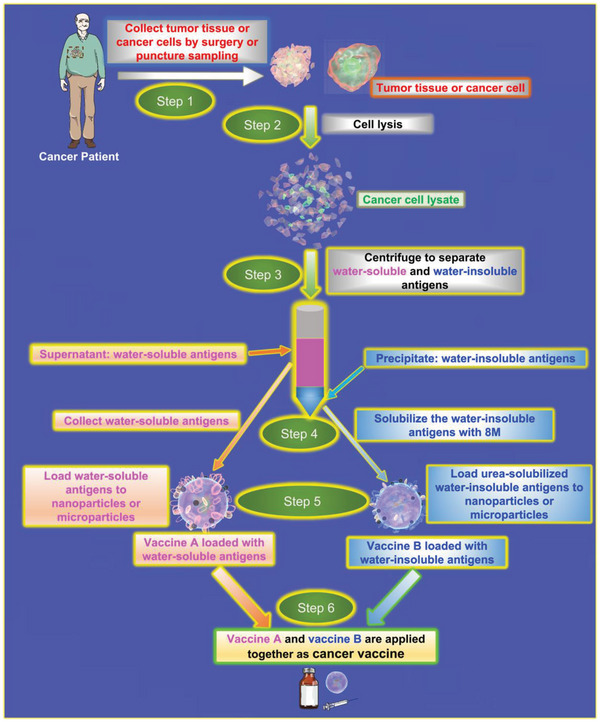
Process of preparing cancer vaccine loaded with both water‐soluble antigens and water‐insoluble antigens of whole cancer cells.

Applying living tumor cells as antigens in cancer vaccine formulation is potentially harmful to patients; in addition, their immunogenicity is relatively poor, which could inhibit DCs maturation and harvest by releasing certain factors. For example, the release of IL‐10 and TGF‐*β* by tumor cells could inhibit DCs and T cell functions.^[^ ^159^ ^]^ Vascular endothelial growth factor (VEGF) released from tumor cells could suppress DCs differentiation and maturation. Soluble major histocompatibility complex (MHC) class I and homologue ligands (MICA and MICB) inhibit NKG2D‐mediated killing by immune cells.^[^ ^4^ ^]^ Therefore, to overcome these issues, it is important to identify the appropriate methods for killing tumor cells and increasing the immunogenicity of tumor antigens. Several methods induce immunogenic death of tumor cells (**Figure** **7**). Controversies are raised on the use of apoptosis and necrosis to extract tumor cell lysates; however, it has been widely applied in several studies. Both apoptosis and necrosis can cause immunogenic death (ICD) of tumor cells, which is associated with the spatiotemporally defined emission of immunogenic DAMPs that can trigger the immune system.^[^ ^160^, ^161^ ^]^ Although the principles of apoptosis and necrosis are different, the obtained cell lysates as antigens pulsed with DCs, which can effectively activate tumor‐specific T cell immunity and improve antitumor therapeutic efficacy. The whole tumor lysate obtained by ICD can fully unleash the antigens, exposing antigen information and promoting a strong specific immune response.^[^ ^162^, ^163^, ^164^, ^165^ ^]^ Several commonly used methods for preparing whole tumor lysates and the methods for designing vaccines to improve the immunogenicity of antigens are discussed below.

Tumor tissue or cell lysate is a cheap and safe source of antigens, containing the full repertoire of the patient's tumor‐specific antigens (neoantigens), that can be obtained by surgically removing the tumor. Several methods have been used to prepare tumor cell lysates; one widely used and straightforward method is the repetitive rapid freeze‐thawing of cancer cells.^[^ ^166^, ^167^ ^]^ This method results in a mixture of all kinds of cellular components including fragments of the destroyed cellular membrane, intracellular organelles such as mitochondria, and cellular RNA and DNA.^[^ ^168^ ^]^ In addition, killing tumor cells by freeze‐thaw lysis exposes normally hidden intracellular molecules such as high mobility group protein (HMGB1), calreticulin, ATP, uric acid, nucleic acids, and lipids, which are recognized by toll like receptors (TLRs) expressed on antigen‐presenting cells (APC). The “danger” and stress signal pathways are activated leading to further activation of immune responses.^[^ ^115^, ^169^ ^]^ During infection, the DNA and RNA in tumor lysates can activate innate immune responses through transmembrane TLRs and cytosolic receptors. Inflammatory signals activate DCs antigen presentation and the migration to secondary lymphoid organs. HMGB1 plays an important role in this process as a chemical attractant for immature DCs, including RAGE. In addition, it has the ability to upregulate the surface proteins, CD80, CD83, and CD86 and stimulate DCs production of the cytokines, IL‐6, CXCL8, IL‐12p70, and TNF‐*α*.^[^ ^170^, ^171^ ^]^ Tumor lysates as antigens can favor the induction of DCs maturation, the release of pro‐inflammatory cytokines, and the improvement of Ag cross‐presentation. In addition, tumor lysates are essential for the priming and activation of a CD8+ T cell‐mediated immune response, resulting in anti‐tumor clinical effectiveness.^[^ ^172^, ^173^ ^]^

Tumor lysates are good antigen resources to prepare cancer vaccines, administered either directly after mixing with adjuvants or after encapsulation into specific formulations.^[^ ^174^ ^]^ Studies have shown that, to some extent, tumor lysate plus adjuvants can activate the immune system. Combination of lyophilized melanoma cell lysates and the adjuvant Detox PC (Corixa‐Montana) was approved for the treatment metastatic disease in Canada in 1999. Some other adjuvants such as cytosine‐phosphodiester‐guanine oligodeoxynucleotide (CpG), TLR agonists, and IFN‐*α*‐2b were also tested. Melanoma lysates combined with IFN‐*α*‐2b extended survival in 700 patients and reduced the toxicity of the combination regimen.^[^ ^175^ ^]^ Michael et al. discovered that CpG/lysate vaccine could enhance cross‐presentation of antigens and significantly increase cytotoxic T lymphocyte (CTL) proliferation.^[^ ^176^ ^]^

Tumor lysates contain self‐antigens that could dampen the immune response. One of the strategies employed in improving immunotherapy for cancer is the use of ex vivo generated DCs pulsed with tumor antigens.^[^ ^177^ ^]^ For example, DCs pulsed with GL261 tumor cell lysates induced an effective immune response and significantly increased the survival of glioblastoma multiforme mice. It can effectively increase the enrichment of CD8+ T cells in tumor sites and regulate the tumor microenvironment by releasing inflammatory factors such as IFN‐*α* and TNF‐*α*.^[^ ^178^ ^]^ Several studies use whole water‐soluble tumor lysates to pulse autologous DCs to treat tumors; this is effective in many cancers, such as melanoma, Neuro‐2a neuroblastoma, glioblastoma multiforme (GBM), renal tumor, and non‐small cell lung cancer.^[^ ^39^, ^179^ ^]^ Supporting the use of whole water‐soluble tumor lysates as cancer vaccine is a prior phase II trial for GBM, which showed an expansion of CD8+ T cells and CTL against tumor antigens. For example, the expansion of HER‐2, MAGE‐1, and GP100 in 4/9 patients and systemic cytotoxic response of peripheral blood mononuclear cells (PBMCs) in 6/10 patients when treated using DCs pulsed with whole water‐soluble tumor lysates.^[^ ^180^ ^]^ In another clinical trial with 43 stage IV patients and 7 stage III melanoma patients, autologous DCs that were pulsed with a novel allogeneic water‐soluble cell lysate (TRIMEL) from three melanoma cell lines were used. More than 60% of the stage IV patients had positively delayed type of hypersensitivity reaction after vaccination.^[^ ^181^ ^]^ In a recent clinical trial, 28 patients received Gliadel wafers and DCs vaccination, among which 25 patients showed elevated IFN‐*γ* responses.^[^ ^180^ ^]^

To upregulate the ability of DCs to present antigens to antigen‐specific T cells, methotrexate was combined with DCs pulsed with tumor cell lysates, to improve T cell priming and proliferation.^[^ ^182^ ^]^ Various TLR ligands, such as LPS, Poly (I:C), and Bacillus Calmette‐Guerin (BCG) were tested for combining with DCs, to improve DCs maturation. These ligands can stimulate the upregulation of the surface co‐stimulatory molecules, CD80, CD86, and CD40, resulting in the release of proinflammatory factors, IL‐12, TNF‐*α*, and IL‐6.^[^ ^183^ ^]^ Cyclophosphamide enhances antitumor efficacy when combined with DCs pulsed with tumor lysates; this can increase the proportion of IFN‐*γ* secreting and decrease the proportion of CD4+CD25+FoxP3+ regulatory T (Treg) cells in the spleen.^[^ ^184^ ^]^ Therefore, ICD of cancer cells is promising when combined with DC‐based vaccines.^[^ ^185^ ^]^

Another popular method of preparing tumor lysates is using UV ray irradiation to obtain apoptotic tumor lysates.^[^ ^186^ ^]^ UV light is considered an electromagnetic nonionizing radiation with a wavelength between 100 and 400 nm. Its immunogenic potential was discovered in 1991 when Begovic et al. demonstrated that UV‐irradiated cancer cells could induce resistance to subsequent rechallenge with cancers.^[^ ^187^, ^188^ ^]^ During apoptosis, the induction of specific DAMPs, such as membrane translocated calreticulin (CRT) and the release of heat shock protein 70 (HSP70) and HMGB1 determines the immunogenicity of UV‐irradiated cancer cells. Several studies have shown that immunization with DCs loaded with UV‐treated tumor cells could elicit effective antitumor therapeutic efficacy in a B16 mouse melanoma model, albeit non‐superior to DCs loaded with necrotic freeze‐thaw lysate.^[^ ^189^ ^]^ UV light can affect the cell's DNA mutation‐induced tumor neo‐antigens, which might also contribute to the increase in host antitumor immune response. A clinical study showed that combined administration of UV‐irradiated autologous cancer cell lysates and BCG as an adjuvant was able to treat 81 stage III and IV metastatic melanoma patients with a 5‐year patient survival rate of approximately 45%. A preclinical study showed that pulsing DCs with UV‐irradiated tumor cell lysates could activate cellular immunity by releasing IL‐12. However, there is no report of a clinical trial that used UV irradiation as the sole treatment for obtaining an antigen source to pulse DCs. This is because UV light treatment alone cannot induce high levels of cancer ICD.^[^ ^190^, ^191^ ^]^

#### 4.1.2 Cancer Vaccines Based on Tumor Lysates Whose Immunogenicity Was Improved with Oxidative Modification

Hypochlorous acid (HOCL) is a strong bactericidal oxidant produced by activated neutrophils in acute inflammation.^[^ ^192^ ^]^ HOCL enhances immunogenicity through protein antigens; in addition, it increases antigen uptake and processing by antigen‐presenting cells as well as activation of antigen‐specific T lymphocytes.^[^ ^193^ ^]^ Early in vitro studies have shown that when T cells are incubated with HOCL‐treated model antigens such as ovalbumin (OVA) or bovine serum albumin (BSA) ‐stimulated APCs, there is an increase in T cell response, including a stronger antigen recognition, faster cell proliferation, and higher IL‐2 production. SKOV‐3 ovarian tumor cells were chosen as the model to study the effect of tumor antigen obtained through HOCL‐oxidation; it induced rapid primary necrosis, enhanced the uptake of ovarian tumor by DCs, and primed autologous tumor‐specific CD4+ and CD8+ T cell responses.^[^ ^194^ ^]^ In a pilot study of five patients with recurrent ovarian cancer, the immunogenicity, clinical efficacy, and progression‐free survival (PFS) of autologous DCs pulsed with HOCl‐oxidized autologous tumor lysate (OCDC vaccine) were evaluated. OCDC vaccine induces an effective T cell response against known ovarian tumor antigens. Circulating T regulatory cells and serum IL‐10 levels were also reduced.^[^ ^101^, ^195^ ^]^ Two subjects had a persistent progression‐free survival of more than 24 months following the OCDC treatment.^[^ ^196^ ^]^ A novel strategy using nanoscale therapeutics showed that treating a mouse breast cancer cell line (EMT6 cells) with 60 µm HOCl for 1 h could ensure complete cell death. HOCl‐oxidized tumor lysates were mixed with CpG and enveloped in liposomal spherical nucleic acids (SNAs) for triple‐negative breast cancer (TNBC). This strategy significantly increased the activation of DCs compared to that using their non‐oxidized counterparts. Simultaneously, the population of cytotoxic CD8+ T cells was increased and the population of myeloid derived suppressor cells (MDSCs) was decreased within the tumor micro‐ environment.^[^ ^197^ ^]^

Some studies have explored the mechanism underlying the effects of HOCl‐treated tumor cell lysates; it appears to be more immunogenic when used as an antigen source for therapeutic DC‐based vaccines. The increased immunogenicity could be attributed to qualitative and quantitative changes in the human leukocyte antigen class II (HLA‐II) ligandome, which potentially leads to an increased HLA‐II dependent stimulation of the T cell compartment.^[^ ^198^ ^]^ Not only DCs but T cells also have been used to study the immune function stimulated by HOCl‐oxidized melanoma cell lysates, but the result was a bit unexpected.^[^ ^199^, ^200^ ^]^ T cells that were stimulated with oxidized melanoma cells were specific to the oxidized melanoma cells and MART‐1 peptides. These studies were designed to test the immunogenicity and safety of oxidized autologous tumor lysates with or without DCs and in combinatorial immunotherapy strategies.^[^ ^201^ ^]^

#### 4.1.3 Cancer Vaccines Based on Tumor Lysates Whose Immunogenicity Was Improved with Heat Shock

Heat shock is a term that is applied when a cell is subjected to a temperature that is higher than the ideal body temperature of the organism from which the cell is derived. Heat shock can induce apoptosis (41−43 °C) or necrosis (>43 °C).^[^ ^202^ ^]^ The mechanism underlying the enhanced immunogenicity of tumor cell lysates under heat shock is the production of heat shock proteins (HSPs). When tumor cells are exposed to high temperatures, they are thermally stimulated to produce HSPs to protect themselves.^[^ ^203^ ^]^ Many heat shock proteins such as HSP110, HSP90, HSP70, HSP60, and small Heat Shock Proteins (sHSPs) have molecular chaperone activity. The HSPs are divided into five subgroups according to their protein size. Stress‐induced HSP‐peptide complexes (commonly induced during apoptosis) are more efficiently taken up via scavenger receptors and TLR on the DCs surface, and they induce efficient cross‐priming and skewing of the immune response toward a TH1‐type profile.^[^ ^204^ ^]^ In vitro, human DCs loaded with melanoma cells that were heat‐treated at 42 °C before being killed showed more efficient cross‐priming against naïve human CD8+ T cells than DCs loaded with unheated melanoma cells. In addition, in vitro‐generated DCs that were stimulated with TRIMEL from melanoma patients induced a fivefold increase in IFN‐*γ* release through a melanoma‐specific cytotoxic T cell clone, compared to APCs that were stimulated with a non‐HS‐treated melanoma cell lysate.^[^ ^205^, ^206^ ^]^

The use of allogeneic heat shock‐conditioned tumor cell lysates provides a vast number of different tumor‐specific antigens and also delivers different DAMPs such as HMGB1 and CRT, which are necessary for the proper maturation, activation, and cross‐presentation of tumor‐associated antigens to DCs, in turn enhancing their antitumor‐induced response. A great deal of research has been done on heat shock‐modified tumor lysates to improve its immunogenicity.^[^ ^207^, ^208^ ^]^ In a recent study, DCs loaded with heat shock‐conditioned tumor cell lysates were successfully applied in clinical practice for high grade glioma patients. Together, these studies reveal that the heat shock‐treated tumor lysates are sufficient to improve antigen immunogenicity and boost the systemic immune response.^[^ ^209^, ^210^, ^211^ ^]^

#### 4.1.4 Cancer Vaccines Based on Tumor Lysates Whose Immunogenicity Was Improved with Virus

Oncolytic viruses are a kind of tumor‐killing viruses with replication ability. The earliest occurrence of oncolytic viruses in the world was reported when a patient with cervical cancer was found to be infected with rabies virus and her tumor subsequently subsided.^[^ ^212^ ^]^ In recent years, oncolytic viruses have been widely studied and used in tumor therapy. Oncolytic virotherapy is an established cancer treatment whereby virus‐based therapies are used for intratumoral application. Preferential lysis of cancer cells leads to the release of danger signals and tumor‐associated antigens, which trigger potent adaptive anti‐tumor responses.^[^ ^213^ ^]^ T‐VEC is an oncolytic herpes simplex virus type 1 (HSV‐1) that attenuates selective cancer cell replication and encodes GM‐CSF to promote local accumulation of DCs and facilitate antitumor immune responses. A study investigated the clinical, molecular, and immunologic effects of oncolytic virotherapy in 13 primary cutaneous B cell lymphoma (pCBCL) patients treated with intralesional T‐VEC in a phase 1 clinical trial. Early influx of therapy‐induced NK cells and monocytes in both injectable and non‐injectable lesions, accompanied by an increase in clonal CD8+ T cells and a decrease in the proportion of Treg helper cells and CD8+ T cells was observed.^[^ ^214^ ^]^ Interestingly, oncolytic virus has the capability to revert a non‐inflamed phenotype into an inflamed phenotype. The virus‐released cancer antigens of OV‐associated molecular patterns, such as viral capsid constituents, proteins, and viral RNA and DNA, stimulate Toll‐like receptors and the danger signals that are necessary to initiate innate and adaptive immunity.^[^ ^215^ ^]^ In addition, oncolytic virus triggers tumor cell ICD by increasing the surface expression of calreticulin and the synthesis of ATP and HMGB1, which trigger immune activity and initiate anti‐tumor immunity.^[^ ^216^, ^217^ ^]^

The tumor microenvironment is often characterized by a lack of tumor‐reactive immune cells; this combined with the tumor itself that initiates a number of immunosuppressive mechanisms, the anti‐tumor immune response often falls short. To enhance its immunotherapeutic effect, sufficient viruses that can release cytokines such as IL‐12, CCL4, and antagonists have been reported in some studies.^[^ ^218^ ^]^ New research is focused on ONCR‐177, which is an engineered recombinant oncolytic HSV with complementary safety mechanisms, including miRNA attenuation and mutant UL37 to inhibit replication, neuropathic activity, and latency in normal cells. DCs that are activated with ONCR‐177 tumor lysates efficiently stimulated tumor‐specific CD8^+^ T cell responses.^[^ ^219^ ^]^

Some progress has been made in the treatment of lung cancer using oncolytic virus in phase I/II clinical trials. The recombinant vaccinia virus, GLV‐1h68 enhanced tumor growth inhibition of human PC14PE6‐RFP lung cancers.^[^ ^220^ ^]^ Advanced prostate cancer (PC)‐specific oncolytic adenovirus is armed with a fusion gene of prostate‐specific antigen and CD40 ligand; the results were groundbreaking. There was an upregulation in the expression of CD80, CD83, and CD86, and the mRNA levels of IL‐6, IL‐12, IL‐23, and tumor necrosis factor‐*α*, significantly.^[^ ^221^ ^]^ Having demonstrated that oncolytic viruses are effective against a variety of solid tumors, the question now is whether they are effective against brain tumors. Oncolytic viruses have very limited ability to spread, cross the host tumor, and penetrate the blood‐brain barrier. These factors limit the clinical effectiveness of oncolytic viruses in treating gliomas.^[^ ^222^ ^]^ However, the study used neural stem cells (NSCs), which can migrate and spread freely within tumors, to deliver a genetically engineered oncolytic adenovirus (CRAd‐S‐pk7) in a first‐in‐human, open‐label, phase 1, dose‐escalation trial. The phase1 trial has proved the safety and tolerability of NSC‐CRAd‐S‐pk7 injection during surgery in patients, considering that the patients had favorable clinical outcomes (in terms of survival). This is the first clinical trial that achieves a dose‐escalation in humans and provides theoretical support for the use of NSCs to deliver engineered oncolytic adenovirus in patients with high‐grade glioblastoma.^[^ ^223^ ^]^

Oncolytic virotherapy as a local treatment would be reserved for highly selective patients, and it could have some limitations in treating the infiltration. Therefore, some new strategies, such as combining a local virotherapy with a systemic adoptive cell transfer therapy, are being tested. For example, some chemically generate a perceptive tumor microenvironment (TME) while others are based on immune modulators and apoptosis‐stimulating drugs. These projects are undergoing clinical trials.^[^ ^224^, ^225^, ^226^ ^]^

#### 4.1.5 Cancer Vaccines Based on Whole Water‐Soluble Components Treated with Formalin

Palacios et al. constructed an autologous vaccine that is composed of the whole water‐soluble components of tumor tissues together with BCG and formalin. At the 5‐year follow‐up, the survival rate reached 60% for the combined treatment.^[^ ^227^ ^]^

#### 4.1.6 Summary of Cancer Vaccines Based on Water‐Soluble Components

Thanks to the presence of a variety of water‐soluble antigens of tumor cells, cancer vaccines based on water‐soluble components can activate broad antigen‐specific immune responses and have showed good efficacy in preventing and treating cancer. The cancer vaccines based on water‐soluble components in tumor cell/tissue lysates, have showed promising efficacy in preventing and treating cancer. However, the efficacy could be further improved by overcoming some limitations. The limitations include but are not limited to:

1. Water‐insoluble antigens generally are more immunogenic and water‐insoluble components, containing a large number of water‐insoluble antigens, are excluded from the vaccine formulation, because of the lack of proper solubilizing methods for solubilizing the water‐insoluble components in tumor cell lysates. Therefore, the therapeutic or preventive efficacy of the cancer vaccine is relatively low.
2. It is difficult for water soluble antigens to be uptaken by APCs and penetrate the cell membrane, which is hydrophobic; therefore, a lot of antigens cannot be efficiently uptake by antigen‐presenting cells (APCs). This further limits the therapeutic or preventive efficacy of cancer vaccines loaded only with water‐soluble components of tumor cell lysates.

### 4.2 Cancer Vaccines Based on Whole Tumor Cell/Tissue Lysates (Containing Both Water‐Soluble and Water‐Insoluble Antigens)

To date, neoantigen‐based cancer vaccines, customized to individual patients, have been widely investigated in the clinic; however, they have not been approved due to various limitations.^[^ ^228^, ^229^ ^]^ One critical limitation is that the antigens loaded in such cancer vaccines is not broader enough and thus cannot induce pan‐clones of tumor‐specific T cells, which is needed to efficiently control the rapid expansion of tumor cells. The use of whole tumor cell lysates to prepare cancer vaccines is one promising approach that obviates some of the challenges in defining specific antigens for vaccine development. Cancer vaccines based on whole tumor cells, can benefit from pan‐spectra patient specific antigen presenting, eliciting immunity against a broad spectrum of tumor‐specific antigens. To our knowledge, previous studies focused on investigating cancer vaccines based on water‐soluble tumor antigens in tumor tissue/cells lysates, due to the difficulty of solubilizing the water‐insoluble components in tumor tissues/cells lysates. However, water‐insoluble neoantigens are more immunogenic, and most components of tumor tissue/cells are water‐insoluble. These contain a large amount of water‐insoluble neoantigens. These water‐insoluble tumor neoantigens may be beneficial for vaccine‐based cancer immunotherapy. Therefore, in Liu's studies, they gently solubilized the water‐insoluble components in tumor tissues/cells lysates using 8 m urea and encapsulated the urea‐solubilized water‐insoluble components into a nanovaccine formulation or microvaccine formulation.^[^ ^37^ ^]^ The process to prepare such nanovaccines or microvaccines are easy (needs 1 day to 4 days to prepare such vaccines), and such nanovaccines or microvaccines only include biocompatible materials, PLGA and whole tumor cell/tissues lysates, which ensure the safety of such vaccines.

Co‐encapsulating whole tumor cell/tissue lysate and immune adjuvants into nanovaccines or microvaccines can deliver antigens and immune adjuvants into the same APC. Therefore, whole tumor cell/tissues lysates provide first signal for activating T cells through the whole tumor antigens, and the immune adjuvants (such as poly (I:C)) provide the second and third signals for activating T cells by inducing co‐stimulating signals and cytokines. All these facilitate the efficient activation of T cells. In addition, APCs prefer to uptake nanoparticles or microparticles; therefore, water‐soluble antigens are uptaken efficiently. This overcomes the uptake and penetrating barrier of water‐soluble molecules in the APC membrane that is lipophilic (Figure 7).

In melanoma and breast cancer, such nanovaccines loaded with whole cell components (including both water‐soluble and water‐insoluble antigens), showed better immunotherapy efficacy than nanovaccines loaded with only water‐soluble components of tumor cell/tissue lysates in mouse cancer models. The combination with *α*PD‐1 antibody and metformin can further improve the efficacy of nanovaccines based on whole tumor cell/tissue lysates.^[^ ^20^ ^]^ In addition, nanovaccines or microvaccines loaded with whole cell components (including both water‐soluble and water‐insoluble antigens), can efficiently prevent and treat lung cancer, melanoma, breast cancer, hepatoma, and cancer metastasis.^[^ ^37^ ^]^

Free cell lysates mixed with adjuvants have been applied as cancer vaccines to treat cancer; however, their efficacy is limited. One important reason is that water‐soluble antigens in tumor cell lysate cannot penetrate the lipophilic membrane of APCs.^[^ ^230^ ^]^ In addition, free cell lysates and immune adjuvants cannot be uptaken or interact with the same APC, which is critical for efficient activation of T cells. APCs preferably uptake nano‐sized or micron‐sized particles; therefore, nanovaccines or microvaccines can be more efficiently phagocytosed by APCs than free cell lysates mixed with adjuvants. The co‐delivery of whole‐cell antigens and adjuvants within APCs using nanovaccines or microvaccines could more effectively activate antigen‐specific T cells than free cell lysates mixed with adjuvants. Nanovaccines or microvaccines provide primary activation signal, and secondary and third activation signals simultaneously in the APCs.

The vaccines‐encapsulated‐whole‐components‐of‐tumor‐tissue (VEWCOTT) can not only effectively prevent or treat the same type of cancer, but can also elicit a strong cross‐cancer specific immune response. Such strong cross‐cancer specific immune response, induced by nanovaccines or macrovaccines (loaded with whole tumor cell/tissue lysates), could be used to prevent or treat cancer in different organs and metastatic cancers, considering that metastatic cancers and recurrent cancers are diverse, different from primary cancers, and are organ‐dependent.^[^ ^37^ ^]^

Proteomics studies demonstrated that many neoantigens are shared between melanoma cells and lung cancer cells. The discovery of antigen sharing among different kinds of cancers and across cancer immune responses among different types of cancers, indicated that cancer vaccines, based on whole tumor cell/tissue lysates, has the advantage of simultaneously preventing cancer metastasis and cancers in different organs.^[^ ^37^ ^]^ However, the existence of a large amount of shared neoantigens among different types of cancers demonstrated that it is critical to include the shared neoantigens in cancer vaccines, either neoantigen‐based cancer vaccines or other formed cancer vaccines. The antigen‐specific immune responses could depend on a large number of neoantigen groups or specific neoantigen group combinations.

In situ cancer vaccines attracted more and more attentions to be applied as therapeutic vaccines, due to its ability to manipulate the cancer cells in tumor tissue. Theoretically, methods that kill cancer cells in tumor tissues and promote the release of tumor antigens from dead cancer cells in tumor tissues can be recognized as in situ cancer vaccines. For instance, oncolytic virus, oncolytic bacteria, particle implantation radiotherapy, and injecting biomaterials or polymers into tumor tissues etc. can all be recognized in situ cancer vaccines, in some extent. These methods can kill cancer cells in situ and stimulate immune responses, relying on cancer cell death in tumor tissue and the release of DAMPs which can activate innate immune cells.

In situ cancer vaccines have some advantages, such as utilizing the cancer cells in tumor tissues, etc. However, in situ cancer vaccines also have some limitations, such as:

1) Most in situ cancer vaccines need intratumoral injection; 2) cannot control the amount of cancer antigen release; 3) cannot be preventatively used as prophylactic vaccines; 4) most dead cancer cells could be uptake by nearby live cancer cells and thus providing nutrients to the live cancer cells; 5) the lack of effective APCs in tumor tissues; 6) APCs uptake antigens in immune suppressive tumor tissues, which may affect the subsequent T cell activation; 7) most released antigens in tumor tissues are water‐soluble, and thus most antigens are difficult penetrate hydrophobic cell membrane and are difficult to be uptake by APCs; 8) the lack of co‐delivering antigens and adjuvants to same antigen‐presenting cell which is needed to activating T cells by simultaneous presence of 3 signals (signal 1: antigen epitopes; signal 2: co‐stimulatory molecules; signal 3: proinflammatory cytokines); 9) could be difficult to inject into small‐sized tumor tissues and so on.

Compared with the in situ tumor vaccine, nanovaccines or micronvaccines loaded with whole tumor cell lysates have the following advantages: 1) promote both water‐soluble and water‐insoluble antigens (encapsulated into nanovaccines or micronvaccines) can be more efficiently uptaken by APCs, thanks to APCs' natural preference to uptake nano‐sized or micron‐sized particles; 2) nanovaccines or micronvaccines are uptaken in injecting site or nearby lymph nodes, but are not uptaken in the immune suppressive tumor tissues, which is beneficial for subsequent T cell activation; 3) can more efficiently activate antigen‐specific T cells by co‐delivering antigens and adjuvants into the same APC, which need the simultaneous presence of 3 signals (antigens provide signal 1 antigen epitopes; adjuvants provide signal 2 co‐stimulatory molecules and signal 3 proinflammatory cytokines), thanks to antigens and adjuvants that are co‐encapsulated in the same nanovaccine or micronvaccine particle; 4) can be injected subcutaneously or intramuscularly, which is more feasible for patients; 5) can control the amount of cancer cell lysates injected every time; 6) can inject multiple times (even after larger tumor tissue disappear), can design the injection time and the course of treatment; 7) can be used as prophylactic cancer vaccines to prevent cancer and so on.

## 5 Conclusions and Outlook

In summary, tumor derived antigens, especially pan‐spectra antigens, are the best sources for developing cancer vaccines. Though pre‐determined neo‐antigens have been comprehensively investigated in the clinic, no neoantigen‐based vaccine has exhibited the ideal efficacy and no neoantigen‐based cancer vaccines has been approved yet. These could be attributed to the following limitations: it is time‐consuming (several months) to prepare neoantigen‐based vaccines, the process is complicated, the predictions of antigen‐epitopes are not accurate, immunogenicity of the antigens is not positively correlated with the abundance of neoantigens, the high heterogeneity of different cancer cells, and the cost is high. Considering that tumor antigens are highly diverse, and it is difficult to identify the effective neoantigens or neoantigen combinations, the concept of using whole cancer antigens, either whole tumor cells or whole tumor cell lysates, to prepare cancer vaccines seems promising. Cancer vaccines based on whole tumor cells or whole tumor cell lysates could be more effective than neoantigen‐based vaccines, considering that such vaccines contain more diverse neoantigens and neoantigen combinations. Besides, cancer vaccines based on whole tumor cells or whole tumor cell lysates are much cheaper, because they do not involve additional processes, such as pre‐determination. Good therapeutic efficacy has been achieved with whole water‐soluble tumor lysates. Currently, scientists have the ability to utilize the whole tumor cell lysate, including both water‐soluble and water‐insoluble components, as antigens in cancer vaccines and acquire better preventive and therapeutic efficacy. We believe that whole tumor lysates‐based vaccine is a promising treatment option and that novel modalities should be investigated to truly identify the immunogenic potential of these cells. Autologous tumor cells are the best antigen source to prepare cancer vaccines, and they carry a variety of specific tumor antigens; therefore, whole tumor cell lysates are an ideal antigen source to be used as antigens in cancer vaccines. Part of the whole tumor cell components, such as whole RNAs or cell membranes, could also be used as antigens in vaccines. These would impart the efficacy of activating some antigen‐specific immune responses. However, it needs to be further investigated in the clinic, especially whether whole tumor cells or tumor cell lysates are indispensable for achieving the best efficacy and whether a part of the whole tumor cell components (such as whole RNAs and membranes) could achieve a similar efficacy. Herein, we summarize the progress of cancer vaccines based on whole tumor cell components (whole tumor cells, whole tumor cell lysates, and whole water‐soluble components in tumor cell lysates) and part of whole tumor cells components (whole RNAs, exosomes, and membranes) in recent years. We believe that this information could help in the translation of the study findings in clinical indications.

## Conflict of Interest

The authors declare no conflict of interest.

## Author Contributions

M.L. conceived and designed the review and revised the paper. L.D. wrote the draft of the review and revised the review.
